# Prof. Darrach’s Introductory Lecture

**Published:** 1847-03

**Authors:** 


### INTRODUCTORY LECTURE ON LIFE. BY PROFESSOR DARRACH.

The Professor of the Theory and Practice in the medical department of *Pennsylvania College*, in selecting a topic for the opening of his course, seems to have assumed that his pupils were further advanced in the study of science than that class of auditors generally is. Its abstruseness is suggested by its very title—Life. After contrasting organic with inorganic bodies, he proceeds to a classification of the former into three divisions—Vegetables, Animals, and Man; the latter deriving his claim to a separation from all the rest, by the possession of a soul. In sketching the characteristics of these divisions, we do not observe anything particularly new; but many parts are executed with spirit, and some passages are eloquent; while, although a few are recondite, and even border on the obscure, none are heavy or unreadable.

